# First record of the subfamily Eucerotinae (Hymenoptera, Ichneumonidae) from the mainland Afrotropics, with a description of a new species

**DOI:** 10.3897/zookeys.1295.212026

**Published:** 2026-10-27

**Authors:** Tapani Hopkins, Alexsandra Nascimento, Bernardo F. Santos, Tomáš Hovorka, Ilari E. Sääksjärvi, Emil M. Österman

**Affiliations:** 1 Zoological Museum, Department of Biodiversity Sciences, University of Turku, 20014 Turku, Finland Zoological Museum, Department of Biodiversity Sciences, University of Turku Turku Finland; 2 Instituto Nacional de Pesquisas da Amazônia — INPA, Programa de Pós-Graduação em Entomologia, Campus II, Caixa postal 478, CEP 69011–97, Manaus, Amazonas, Brazil Instituto Nacional de Pesquisas da Amazônia — INPA, Programa de Pós-Graduação em Entomologia, Campus II Manaus Brazil; 3 Museum für Naturkunde, Leibniz Institute for Evolution and Biodiversity Science, Center for Integrative Biodiversity Discovery, Invalidenstraße 43, Berlin, 10115, Germany Museum für Naturkunde, Leibniz Institute for Evolution and Biodiversity Science, Center for Integrative Biodiversity Discovery Berlin Germany; 4 Department of Entomology, Natural History Museum, National Museum of the Czech Republic, Cirkusová 1740, CZ-193 00 Praha 9 - Horní Počernice, Prague, Czech Republic Department of Entomology, Natural History Museum, National Museum of the Czech Republic Prague Czech Republic

**Keywords:** Barcoding, biogeography, Cameroon, *
Euceros
*, ichneumonoid wasps, mainland Africa, taxonomy, Uganda

## Abstract

The ichneumonid subfamily Eucerotinae has been thought to be almost absent from the tropics, with the only known Afrotropical species found in Madagascar. We report the subfamily as present in the mainland Afrotropics and describe a new species, *Euceros ibex* Hopkins, Nascimento & Santos, **sp. nov**., from Uganda and Cameroon. The subfamily was likely not observed in the mainland Afrotropics before because of low abundances and insufficient sampling. More Eucerotinae likely remain to be discovered in tropical Africa and Asia, although tropical America may genuinely have few eucerotine species. Much more extensive sampling will be needed before it is possible to make confident estimates of how eucerotine diversity is distributed globally.

## Introduction

Eucerotinae is a small subfamily of Ichneumonidae (Hymenoptera) consisting of 51 species in the genus *Euceros* Gravenhorst, 1829 and one species in the genus *Barronia* Gauld & Wahl, 2002 (Gauld and Wahl 2002; Riedel 2018, 2025; Dal Pos et al. 2026). The subfamily’s known distribution is mainly outside the tropics, especially in northern temperate regions and temperate Australia (Gauld and Wahl 2002; Dal Pos et al. 2026). Only a single species of Eucerotinae has been recorded from tropical America, *E. limatus* Barron, 1978 from southeastern Brazil (Barron 1978; Dal Pos et al. 2026). South America also has a non-tropical species, *Barronia araucaria* Gauld & Wahl, 2002 from Chile (Gauld and Wahl 2002; Dal Pos et al. 2026). There are also two eucerotine specimens collected in 1964 which are either a further Chilean species or *B. araucaria* (Porter 1997, their current location is unknown). Only a single species is currently known from tropical Africa, *E. madecassus* Seyrig, 1934 from Madagascar (Dal Pos et al. 2026). The only other known African species is *E. tunetanus* (Schmiedeknecht, 1900) from North Africa (Barron 1978; Dal Pos et al. 2026). Tropical Asia has two species recently recorded from Laos, *E. kiushuensis* Uchida, 1958 and *E. rufocinctus* (Ashmead, 1906) (Riedel 2025), and some species, such as *E. latitarsus* Barron, 1978 whose distribution may stretch into the northernmost parts of tropical Asia (Dal Pos et al. 2026). Otherwise, tropical Asia is devoid of Eucerotinae observations, although recent collecting in Thailand (Quicke et al. 2023) appears to have caught one Eucerotinae of a likely undescribed species (identifier THAMK10015–23 in Anon 2023; GBIF.org 2025a).

The biology of the subfamily is highly unusual and, as far as is known, unique for Ichneumonidae, resembling that of the hymenopteran family Trigonalidae (Carmean 1991). Females lay stalked eggs on plants, from which the larvae attach to passing caterpillars or sawfly larvae (Tripp 1961; Gauld and Wahl 2002; Shaw 2014). If or when the host is parasitised by some other ichneumonoid wasp, the eucerotine larva enters the ichneumonoid larva and develops inside it as a hyperparasitoid.

The systematic position of the subfamily is unclear. It has at different times been proposed to be related to, or even part of, subfamilies Lycorininae, Tryphoninae, Ctenopelmatinae, Metopinae, Microleptinae or Brachycyrtinae, but with little convincing evidence for any of these except Brachycyrtinae, with which it is currently believed to be related (Gauld and Wahl 2002; Quicke 2015: 582; Santos 2017; Broad et al. 2018).

In this work, we report the subfamily to be present in the Afrotropics of mainland Africa, in at least Uganda and Cameroon, and describe a new species of *Euceros*. We also discuss the subfamily’s biogeographical distribution.

## Materials and methods

The Ugandan material comes from the “Uganda 2014–2015” collecting event (Hopkins et al. 2019a, 2019b). In that sampling effort, 34 Malaise traps were kept for a year by one of the authors (Tapani Hopkins) in Ugandan forest and farmland, collecting 876 roughly two-week samples of flying insects, with a total sampling effort of 382.4 trap-months. The ichneumonoid wasps (families Ichneumonidae and Braconidae) have been separated from the other insects, and a few selected subfamilies pinned. Four Eucerotinae were separated from the samples and pinned during the processing of other subfamilies. The majority of Eucerotinae remain unprocessed, mixed with other ichneumonoid wasps, and will not be available for study for years. The pinned Eucerotinae are databased in the Finnish Biodiversity Information Facility (https://laji.fi/en), and their data in its current form is also stored in the Zenodo repository (Hopkins et al. 2026).

The Cameroonian material consists of two females, one of which was collected by one of the authors (Tomáš Hovorka) during a two-year (2024–2026) collecting event in southern Cameroon, with twelve Malaise traps in secondary forest near the village of Ebogo. All samples were checked for Eucerotinae. One female specimen (NMPC THNMP000391) was found in trap M2, in a forest clearing between two forest edges. Examination of museum material revealed another, older, Cameroonian specimen in the Natural History Museum, London (NHMUK 010888236).

To obtain DNA barcoding sequences for our new species, we bioinformatically mined genomic data generated from the sequencing of ultraconserved elements (UCEs, Faircloth et al. 2012) as part of an ongoing study on the phylogeny of Ichneumonidae (Santos et al. in prep). Sequences for the Folmer fragment of the COI gene (Folmer et al. 1994) were pulled from the assembled reads using the script assembly_match_contigs_to_barcodes from the Phyluce v. 1.5 pipeline (Faircloth 2016).

We took focus-stacked photographs of the Ugandan holotype specimen with a Canon EOS 7D Mark II digital camera attached to an Olympus SZX 16 stereomicroscope. The programs QuickPHOTO MICRO 3.1 and Deep Focus 3.4 (by PROMICRA) handled the taking of individual photographs, and Helicon Focus 7.7.5 (by Helicon Soft Ltd.) combined them into a focus-stacked image.

We measured wing length from the photographs used for focus stacking using QuickPHOTO MICRO 3.1. We got other measurements by viewing through the microscope, or measuring from the focus-stacked images. For face width versus height, width was the minimum distance between the eyes, and height the vertical distance from the apical margin of the clypeus to the insertion of the antennae.

To verify our African species was new, we compared it to photographs of the holotype of the geographically nearest known species, *Euceros madecassus* in Madagascar (MNHN-EY-EY9555: MNHN and Hervé 2014), photographs of a paratype and non-type specimen of *E. madecassus* (both from RMCA, without identifiers: van Noort 2025), to a non-type specimen of *E. madecassus* from the Natural History Museum, London (NHMUK 010887094), to the morphological codings of Eucerotinae in Gauld and Wahl (2002), and to COI barcodes we found via GBIF (GBIF.org 2025b) or in the Barcode of Life Database (BOLD; https://boldsystems.org/). The two most relevant COI barcodes found this way of *E. madecassus*, came from the Figshare repository (Miraldo et al. 2024, described in Miraldo et al. 2025) and GenBank (accession number JF963331.1, Sayers et al. 2025, described in Quicke et al. 2012) and are included in the dataset of this paper (Hopkins et al. 2026). We used objective clustering (Meier et al. 2006, https://github.com/asrivathsan/obj_cluster) to compare sequence divergence of the COI barcodes.

Morphological terminology follows that of Broad et al. (2018) and Goulet and Huber (1993). The subfamily and generic diagnoses are largely based on Gauld and Wahl (2002). We wrote the diagnoses following the style outlined by Borkent (2021): short, clear diagnoses, which make it easy to confirm the wasp you are holding belongs to the taxon in question.

### Repositories

**NHMUK** Natural History Museum, London, UK

**NMPC** National Museum of the Czech Republic

**RMCA** Royal Museum for Central Africa, Tervuren, Belgium

**ZMUT** Zoological Museum of the University of Turku, Finland

## Results

### DNA barcoding

Objective clustering revealed that the Ugandan and Cameroonian specimens assigned here to *Euceros ibex* sp. nov. differ by 3.67% in their COI sequences. This value exceeds the threshold commonly applied in insect DNA barcoding studies for preliminary species delimitation (e.g. Hebert et al. 2003; Cheng et al. 2023). However, the available material does not provide additional evidence supporting the recognition of two distinct species. Morphological differences between the populations are minor and inconsistent, and the Cameroonian lineage is currently represented by only two partially damaged specimens. Given the limited sampling and absence of clear diagnostic morphological characters, we conservatively interpret the Ugandan and Cameroonian specimens as representing a single species pending the availability of additional material. Future sampling across mainland African populations may help clarify whether the observed COI divergence reflects intraspecific geographic structure or species-level differentiation.

The two specimens of *E. madecassus* differed by only 0.75% in their COI sequences, while differing by over 10% from our newly described species. No other COI sequences available on BOLD had less than 8.28% divergence from our new species.

### Key to Afrotropical species of Eucerotinae

**Table:** 

1	Malagasy species. Metasomal tergites 3+ with lateral black spots	***Euceros madecassus* Seyrig, 1934**
–	Mainland Afrotropical species. Metasomal tergites 2+ with lateral black spots, or spots absent	**2**
2	Tergites 2+ with lateral black spots (Fig. 1B). [Mediodorsal lobe of pronotum with distinct concave notch, which can be hard to see (cf. Fig. 2B).]	***Euceros ibex* sp. nov. (Ugandan specimens)**
–	Tergites without black spots (Fig. 3B). [Mediodorsal lobe of pronotum with shallower concave notch, which can be hard to see (cf. Fig. 2B).]	***Euceros ibex* sp. nov. (Cameroonian specimens)**

### Taxonomy

**Family Ichneumonidae Latreille, 1802**

#### Subfamily Eucerotinae

Taxon classificationAnimaliaHymenopteraIchneumonidae

Viereck, 1919

A57A523F-57AE-5463-934E-0CD33ECAA94B

##### Type genus.

*Euceros* Gravenhorst, 1829.

##### Diagnosis.

**Figure 1.**
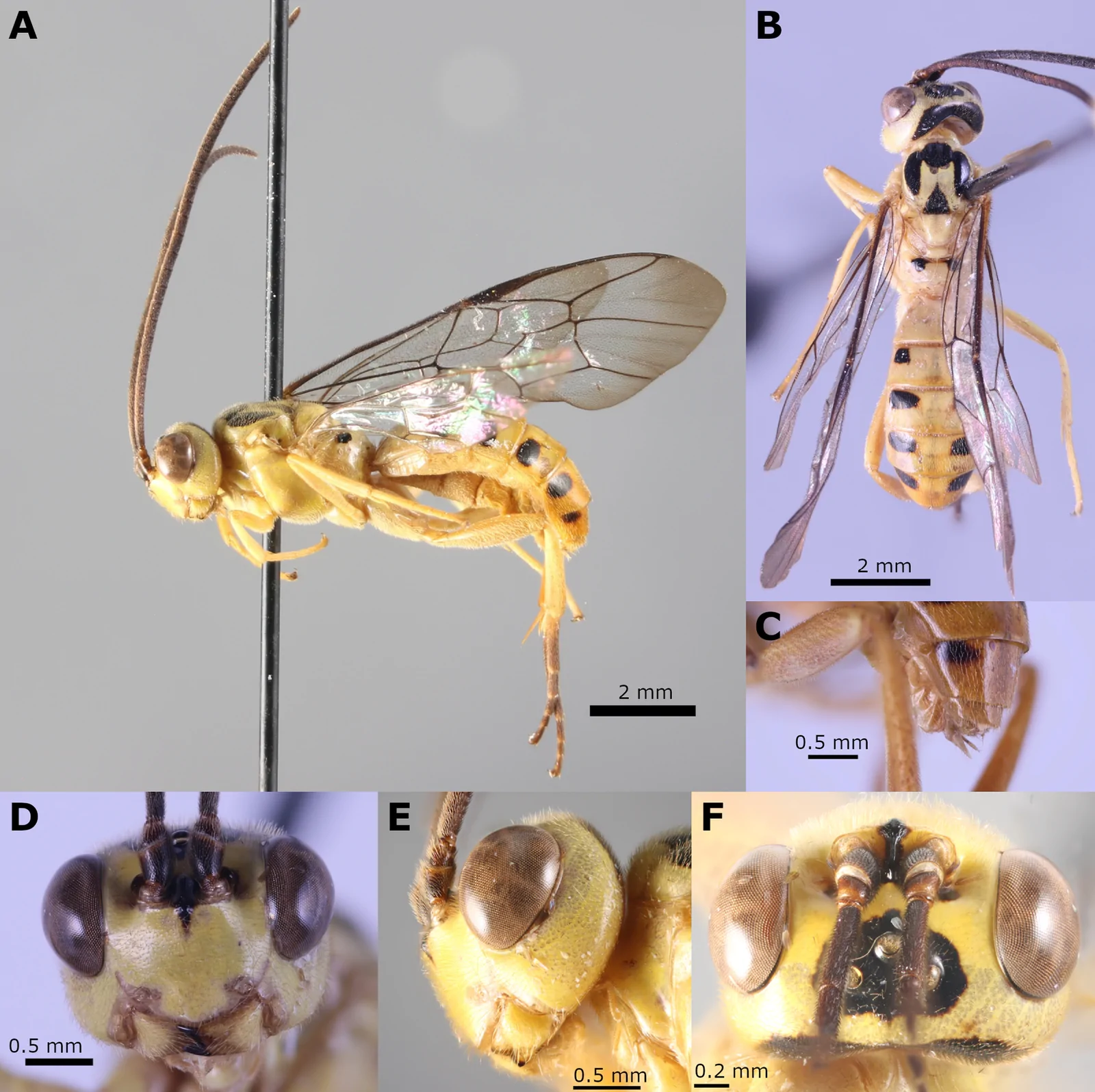
*Euceros ibex* sp. nov. holotype female (http://mus.utu.fi/ZMUT.28283). **A**. Habitus, lateral view; **B**. Habitus, dorsal view (picture from before the left fore wing came loose); **C**. Tip of metasoma, including the ovipositor; **D**. Head, frontal view; **E**. Head, lateral view; **F**. Head, dorsal view.

Areolet open with no trace of wing vein 3rs-m, spiracle of tergite 1 anterior to centre, anterior tergites wide. Female with antennae often centrally slightly flattened and widened, ovipositor tiny and weakly sclerotised (c.f. Fig. 1C). Male with antennae centrally flattened and/or widened, often strikingly so.

##### Remarks.

In practice, the unusual ovipositor of the females and the antennae of the males, together with the distinctive robust habitus (especially the stout tergites), make Eucerotinae relatively easy to recognise. The female ovipositor is so vestigial, they may be mistaken for males at a quick glance. We have found the pectinate hind claws of Eucerotinae a useful additional character: some African male Acaenitinae, for example, can be surprisingly easy to confuse with female Eucerotinae but lack pectinate claws.

#### Genus Euceros

Taxon classificationAnimaliaHymenopteraIchneumonidae

Gravenhorst, 1829

084C28CB-9735-5763-85C0-A5C8A2B6F855

Euceros Gravenhorst, 1829: 328. Type species: Euceros crassicornis Gravenhorst, 1829. Monotypic.
Eumesius Westwood, 1840. 59, 153. Proposed new name for Euceros, due to similarity to Eucera Scopoli, 1770.
Omaloceros Giraud, 1857: 163. Proposed new name for Euceros, due to similarity to Eucera Scopoli, 1770.
Tautozelus Förster, 1869: 212. Synonymised with Euceros by Perkins (1962: 457).
Pseudasthenara Uchida, 1930: 276. Synonymised with Euceros by Townes and Townes (1951: 321).
Lentocerus Dong & Naito, 1999: 210. Synonymised with Euceros by Broad (2021).

##### Diagnosis.

Convex or sometimes truncate apical margin of clypeus (cf. 2B), unlike the only other genus of subfamily Eucerotinae, *Barronia* Gauld & Wahl, 2002, where it is concave (cf. fig. 6 Gauld and Wahl 2002). Clypeal sulcus between clypeus and face absent or weakly impressed in most species, unlike in *Barronia* where it is strongly impressed.

##### Remarks.

Currently, all Eucerotinae except for the one Chilean species *Barronia araucaria* are in the genus *Euceros*.

#### Euceros ibex

Taxon classificationAnimaliaHymenopteraIchneumonidae

Hopkins, Nascimento & Santos sp. nov.

830BCF74-9CCF-53B6-AD14-AD11C11D0DAC

https://zoobank.org/85444DD9-D631-4767-A0BA-78BDB3BB20FA

**Figure 2.**
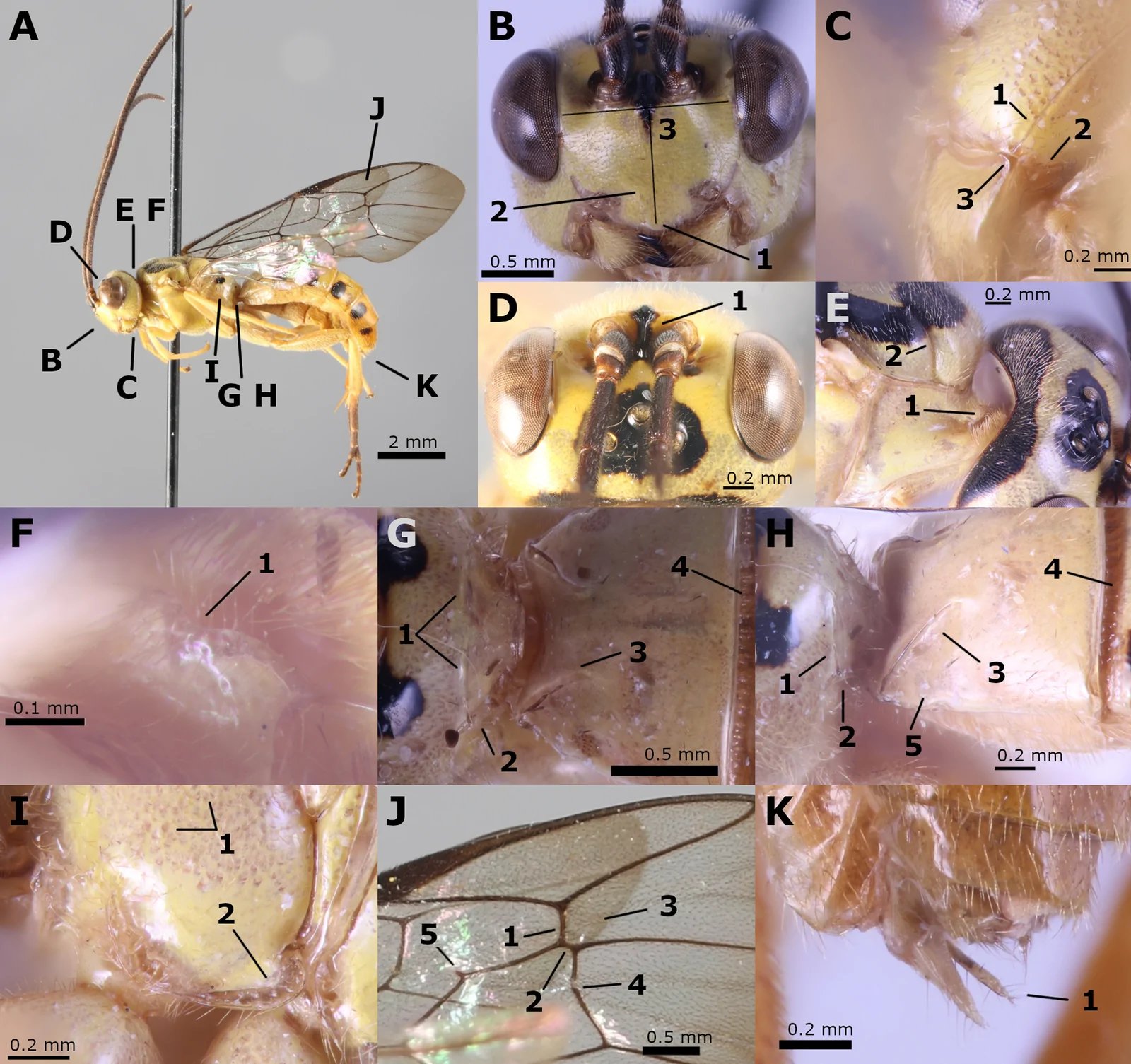
Characters of *Euceros ibex* sp. nov. All images are of the holotype female (http://mus.utu.fi/ZMUT.28283). **A**. Habitus in lateral view, showing where panels B–K are from; **B**. Head in frontal view, showing the convex clypeus margin (1), lack of a clear clypeal sulcus (2), and the width and height of the face (3); **C**. Lower head in lateroventral view, showing that the occipital carina (1) and the hypostomal carina (2) do not merge before the base of the mandible (3); **D**. Head in dorsal view, showing the ridge between the antennal sockets (1); **E**. Anterior mesosoma in dorsolateral view, showing the pronotal lobe (1) and the notauli (2); **F**. Pronotum lobe, showing the mediodorsally concave edge (1); **G**. Propodeum and tergite 1 in dorsal view, showing the weak posterior transverse carina (1), the short lateral longitudinal carina (2), the lateromedian carina of tergite 1 (3), and the anterior transverse groove of tergite 2 (4); **H**. Propodeum and tergite 1 in dorsolateral view, showing the weak posterior transverse carina (1), the short lateral longitudinal carina (2), the lateromedian carina of tergite 1 (3), the anterior transverse groove of tergite 2 (4), and the spiracle of tergite 1 (5); **I**. Propodeum and metapleuron in lateral view, showing the lack of a pleural carina (1), and the submetapleural carina anteriorly expanded into a lobe (2); **J**. Fore wing, showing that 2rs-m (1) is much longer than the segment (2) between it and 2m-cu, the lack of 3rs-m (3), the one bulla in 2m-cu (4), and the ramulus (5); **K**. Tip of metasoma, showing the small weakly sclerotised ovipositor (1).

**Figure 3.**
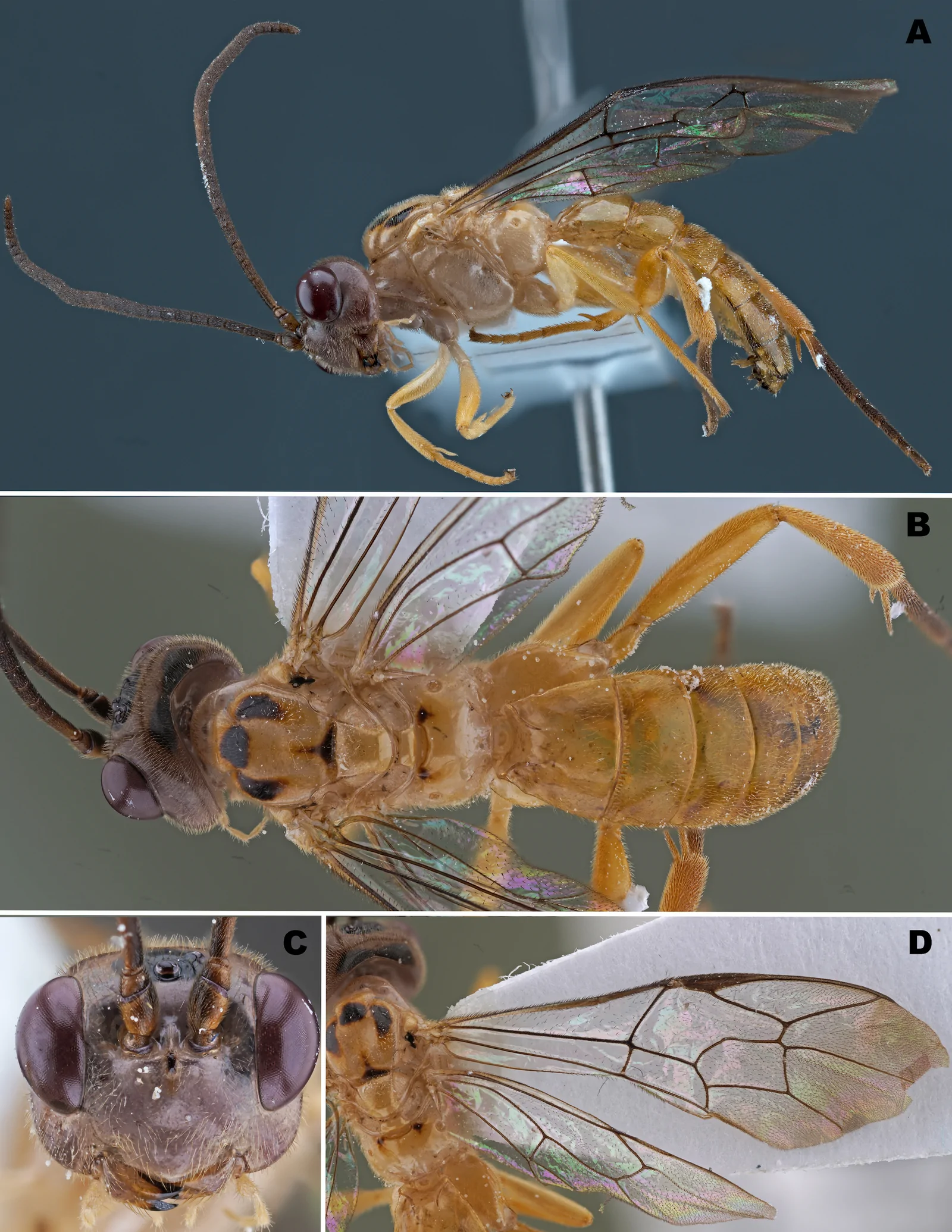
*Euceros ibex* sp. nov. Cameroonian female specimen THNMP000391. **A**. Habitus, lateral view; **B**. Habitus, dorsal view; **C**. Head, frontal view; **D**. Wings. (Scale bar unavailable.)

Figs 1A–F, 2A–K, 3A–D

##### Material examined.

Six females, basic data here and complete data in the Zenodo repository (Hopkins et al. 2026).

***Holotype***. Uganda • ♀; Kibale National Park, Malaise trap R01T1; 0.5532°N, 30.3592°E; 13–28 Aug. 2015; Tapani Hopkins leg.; ZMUThttp://mus.utu.fi/ZMUT.28283.

***Paratypes***. Uganda • ♀; Kibale National Park, Malaise trap R98T2; 0.5532°N, 30.3579°E; 13–28 Aug. 2015; Tapani Hopkins leg.; ZMUThttp://mus.utu.fi/ZMUT.3188 • ♀; Kibale National Park, Malaise trap R93T1; 0.5653°N, 30.3568°E; 7–14 Sep. 2015; Tapani Hopkins leg.; ZMUThttp://mus.utu.fi/ZMUT.26636 • ♀; Kibale National Park, Malaise trap K13T1; 0.5932°N, 30.3598°E; 10–27 Aug. 2015; Tapani Hopkins leg.; GenBank: PZ868595; ZMUThttp://mus.utu.fi/ZMUT.28249.

##### Other material.

Cameroon • ♀; Ebogo, Malaise trap 2; 3.3917°N, 11.4706°E; 6 Oct. 2024; GenBank: PZ868596; NMPC THNMP000391 • ♀; Nkoemvon; 2.815°N, 11.132°E; 1 Aug–30 Sep 1980; NHMUK 010888236.

##### Diagnosis.

**Female**: Only known mainland Afrotropical *Euceros* species. The lateral black spots of the metasoma may be a useful additional character to tell it apart from the Malagasy species *E. madecassus* (but see Remarks). **Male**: unknown, but likely to share the same diagnostic characters, since other *Euceros* species are not known to differ strongly between sexes except for the antennae.

##### Description.

**Holotype** female http://mus.utu.fi/ZMUT.28283. Fore wing length 8.7 mm.

***Head***. Mandibles stout, with dorsal tooth slightly longer than ventral. Margin of clypeus convex when viewed from the front (Fig. 2B). Clypeal sulcus between clypeus and face not impressed, with no clear border between the clypeus and the rest of the face (Fig. 2B). Face about 1.3–1.4 times as wide as high (Fig. 2B). Occipital carina and hypostomal carina reach the base of the mandibles without merging (Fig. 2C). Ridge-like carina in between antennal sockets (Fig. 2D). Antennae with 33 flagellomeres. Antennae very slightly flattened and broadened near centre.

***Mesosoma***. Pronotum with a mediodorsal lobe (Fig. 2E), the apex of which has a concave notch (Fig. 2F). Epomia absent. Mesoscutum smooth with fine hairs, with strongly impressed notauli (Fig. 2E). Mesopleuron smooth, with sternaulus absent and with epicnemial carina reaching above level of lower corner of pronotum. Propodeum smooth; anterior transverse carina absent; posterior transverse carina present in between lateral longitudinal carinae, but very weak (Fig. 2G, H); lateral longitudinal carinae present posterior to the posterior transverse carina, absent otherwise (Fig. 2G, H). Pleural carina absent, so there is no clear border between the propodeum and metapleuron (Fig. 2I). Submetapleural carina anteriorly expanded into a rounded lobe (Fig. 2I). Legs with tarsal claws pectinate.

***Wings***. Fore wing with 2rs-m long and very close to 2m-cu, so that the segment of M between the two veins is much shorter than 2rs-m (Fig. 2J). 3rs-m absent (Fig. 2J). 2m-cu with one bulla (Fig. 2J). 1m-cu&M with a very short ramulus (Fig. 2J).

***Metasoma***. Spiracle of tergite 1 anterior to centre (Fig. 2H). Tergite 1 smooth, with lateromedian carinae stretching from the anterior corners diagonally towards the middle, to about 0.4 times the length of the tergite (Fig. 2G, H). Tergite 2 with an anterior transverse groove (Fig. 2G, H), similar grooves on at least tergites 3–4, although not as easily visible due to being covered by preceding tergite. Ovipositor very small and weakly sclerotised (Fig. 2K).

***Colour***. General colour yellow. Black mandibular teeth, area between antennal sockets, interocellar area, vertex, two median spots anterior and posterior on mesoscutum and two lateral stripes on mesoscutum, two lateral spots on anterior propodeum, lateral spots on tergites 2–6. Antennae dark brown. Hind tarsi dark brown. Wings hyaline but with denser hairs making the forewings slightly darker apically.

##### Remarks.

*Euceros ibex* sp. nov. is easy to recognise since there are only two other known African species, of which *E. madecassus* is likely confined to Madagascar. The other African species, *E. tunetanus*, from North Africa outside the Afrotropics, is mostly black (Barron 1978), and has clear morphological differences (e.g., face densely punctate or granulate, as opposed to polished: Schmiedeknecht 1900; Gauld and Wahl 2002). In the material we have examined, *E. ibex* sp. nov. also differs from *E. madecassus* in the lateral black spots of the metasoma: the spots are present from tergite 2 onwards in the Ugandan material and absent in the Cameroonian material, whereas *E. madecassus* has the spots from tergite 3 onwards. However, when comparing specimens to *E. madecassus* it should be noted that some of the available information about that species is contradictory. Gauld and Wahl (2002) claim it has vestigial notauli and that the submetapleural carina is not expanded into a lobe, and a photograph of the holotype shows no submetapleural lobe (MNHN and Hervé 2014); yet notauli are clearly visible in photographs of the holotype and other specimens (MNHN and Hervé 2014; van Noort 2025), photographs of other specimens seem to show a submetapleural lobe (van Noort 2025), and the non-type specimen we examined has a submetapleural lobe. These may be data errors or unclear photographs, but we have a sneaking suspicion several (likely undescribed) species are involved and are being misidentified as *E. madecassus*.

The Ugandan and Cameroonian specimens of *E. ibex* sp. nov. differ substantially in their COI barcode (3.67%), and show milder differences in morphology and colour. We suspect they may be two separate species, but do not feel confident enough to split the species until more material can be examined.

##### Variation.

The posterior transverse carina of the propodeum varies from very weak to missing in the Ugandan specimens, and is very weak in both Cameroonian specimens. The number of flagellomeres varies from 31 to 33 in the Ugandan specimens (http://mus.utu.fi/ZMUT.28249 not examined), and is 31 in the Cameroonian specimen THNMP000391 (the other Cameroonian specimen NHMUK 010888236 has broken antennae). The fore wing length of the Cameroonian specimen THNMP000391 is 9.4 mm (cf. Ugandan holotype http://mus.utu.fi/ZMUT.28283 8.7 mm; other specimens not measured). The ramulus in the fore wing varies from vestigial to absent in the Ugandan specimens, and is absent in Cameroonian specimen THNMP000391 (other Cameroonian specimen NHMUK 010888236 not examined).

The Ugandan specimens have a clearer concave notch on the mediodorsal lobe of the pronotum than the Cameroonian specimens, whose lobe is very shallowly incised, if at all. However, this character is very hard to see accurately on some specimens.

The general colour varies from bright yellow (the holotype http://mus.utu.fi/ZMUT.28283, and paratype http://mus.utu.fi/ZMUT.3188), to faded whitish yellow with the tip of the metasoma bright yellow (paratypes http://mus.utu.fi/ZMUT.26636, http://mus.utu.fi/ZMUT.28249), to dark yellow (Cameroonian specimens, cf. Fig. 3). The head of Cameroonian specimen THNMP000391 is a burnt ochre in colour, the other Cameroonian specimen NHMUK 010888236 bright yellow. We suspect much of this colour variation may be due to fading or preservation (e.g. THNMP000391 had the inner tissues digested for DNA extraction), and that the holotype is closest to the natural colour.

The Ugandan specimens have lateral black spots on tergites 2 onwards (Fig. 1B), while the Cameroonian specimens lack these spots (Fig. 3B).

##### Etymology.

The specific epithet refers to the shape of the antennae, which resemble the horns of an ibex goat, and is a noun in apposition. The name was suggested by readers of the first authors local newspaper, Österbottens Tidning, who in 2026 sent in name suggestions and voted among five of the suggested names.

##### Holotype condition.

The left fore wing of the holotype is loose.

## Discussion

It is obvious that the subfamily Eucerotinae, despite earlier assumptions to the contrary (Gauld and Wahl 2002), is widespread in mainland tropical Africa. We found a new species in tropical forest in both Uganda and Cameroon; it is exceedingly unlikely that the subfamily would be absent in, for example, the Democratic Republic of the Congo, between these two countries. The mainland Afrotropical species (which may in fact be two separate species) is clearly closely related to the Malagasy species, and the ancestors of the Afrotropical species have presumably either spread from Madagascar to the mainland or vice versa. If Gauld and Wahl (2002) are correct that eucerotines have a Gondwanan origin and spread to the northern hemisphere from the Malagasy/Indian tectonic plate, a Malagasy origin is more likely. However, assumptions of a Gondwanan origin for both Eucerotinae and the possibly related subfamily Brachycyrtinae were partly based on the belief they are absent from the mainland Afrotropics (Gauld and Wahl 2002), which we now know is incorrect for both subfamilies (cf. Di Giovanni and Varga 2021 for Brachycyrtinae).

The lack of earlier observations of this subfamily from the mainland Afrotropics likely reflects its low abundances and insufficient sampling. It will be many years before we know how common the new species is in our Ugandan material, but a rough estimate based on what little material has been processed suggests tens of individuals rather than hundreds in total, collected in 876 samples over a whole year. As a comparison, some of our common Ugandan species (e.g., *Echthromorpha agrestoria* (Swederus, 1787)) number over a thousand individuals. Collecting in non-tropical Georgia, USA also recorded very low abundances of Eucerotinae (Gaasch et al. 1998), nor do we know of any material where Eucerotinae would be abundant. Low abundances are likely the norm for this subfamily throughout its distributional range, perhaps as a result of the family’s highly specialised biology. Such low abundances simply cannot be expected to be detected with the smaller-scale sampling which has largely been the norm in Africa up to now (e.g., van Noort et al. 2000; van Noort 2004, which caught no Eucerotinae).

At a global scale, the presumed near-absence of the subfamily Eucerotinae in the tropics (Gauld and Wahl 2002) seems doubtful. The known diversity of the subfamily is still strongly focused on the northern temperate regions and temperate Australia, but we have discovered a species in tropical mainland Africa, and an undescribed species appears to have recently been found in tropical Asia (Quicke et al. 2023; identifier THAMK10015–23 in Anon 2023). More extensive collecting with numerous Malaise traps played a major part in both these discoveries. It thus seems likely that many more species remain undiscovered in tropical Africa and Asia, and that further collecting is needed before we can tell what the true eucerotine diversity of these regions is. The case for a near-absence of eucerotines in tropical America, on the other hand, is stronger. The only known tropical American species is from southeastern Brazil in high-altitude Atlantic Forest (20°S: Barron 1978; Dal Pos et al. 2026). No eucerotines were caught in Peruvian and Ecuadorian Amazonia or the tropical Andes, in extensive collecting with Malaise traps, host rearing, and canopy fogging that matched our Ugandan collecting in intensity (Sääksjärvi et al. 2004; Gómez et al. 2015; Gómez et al. 2017; summarised in Veijalainen et al. 2013; Hopkins et al. 2024), the material of which is at the Zoological Museum of the University of Turku. Nor did Gauld (1991) report the subfamily Eucerotinae in Costa Rica, despite one of the most intense tropical Malaise trapping and host rearing programmes ever conducted. However, we do not feel the current evidence rules out the possibility of further collecting discovering more tropical American species, merely that a diverse eucerotine fauna seems implausible. The overall picture suggested by the new tropical discoveries is that, while the subfamily Eucerotinae may be more diverse in the temperate areas than the tropics, there is currently too little data to make confident estimates of its global distribution.

Although there is not enough data, the currently most plausible biogeographic distribution for Eucerotinae is that is has the most species in northern temperate regions and temperate Australia, fewer species in tropical Asia and Africa, and almost no species in tropical America. If further collecting confirms this pattern, we speculate that part of the reason for it, beyond the obvious host availability (e.g., few sawfly species in many tropical regions), could be one of predation pressure or competition from the biologically similar Trigonalidae. The unusual biology of eucerotines, with larvae exposed on leaves while waiting for their host, could make them sensitive to predation by e.g., ants, which would drop abundances and hence species richness in high predation environments. Predation pressure is generally highest in the tropics at low elevations (Roslin et al. 2017), which is exactly the kind of habitat in western Amazonia in which we have failed to discover eucerotines. This speculation could potentially be tested by examining the distribution of tachinid fly diversity, since tachinids are much more diverse than Eucerotinae and many species also lay eggs some distance from potential hosts (Stireman et al. 2006). Another, alternative, speculation is that the subfamily Eucerotinae and family Trigonalidae (which both have similar biologies) compete for the same hosts, and that Trigonalidae outcompetes Eucerotinae in the tropics. This would match the currently known distribution of Trigonalidae diversity, which is mainly tropical and subtropical (Carmean and Kimsey 1998).

## Supplementary Material

XML Treatment for Eucerotinae

XML Treatment for Euceros

XML Treatment for Euceros ibex

## Additional information

### Artificial Intelligence (AI) use

The authors accept full responsibility for the content of the manuscript, including the disclosure of any use of AI.

No AI tools were used in the preparation of this manuscript.

### Author ORCIDs

Tapani Hopkins https://orcid.org/0000–0002-2256–0098

Alexsandra Nascimento https://orcid.org/0000–0001-9404–1564

Bernardo F. Santos https://orcid.org/0000–0002-2634–3066

Tomáš Hovorka https://orcid.org/0000–0002-6887–2644

Ilari E. Sääksjärvi https://orcid.org/0000–0002-8107–5607

Emil M. Österman https://orcid.org/0009–0008-8307–5804

### Data availability

The data of the wasps we examined is in the Zenodo repository (Hopkins et al. 2026, https://doi.org/10.5281/zenodo.23035090). The repository also has the COI barcode sequences we used, and the character state data matrix of Gauld and Wahl (2002) updated with the data of our new Euceros species.
